# A simulation study of gene-by-environment interactions in GWAS implies ample hidden effects

**DOI:** 10.3389/fgene.2014.00225

**Published:** 2014-07-21

**Authors:** Urko M. Marigorta, Greg Gibson

**Affiliations:** Center for Integrative Genomics, School of Biology, Georgia Institute of TechnologyAtlanta, GA, USA

**Keywords:** gene-by-environment, environmental perturbation, modern lifestyle, complex disease, genetic risk score, decanalization, GWAS, obesity

## Abstract

The switch to a modern lifestyle in recent decades has coincided with a rapid increase in prevalence of obesity and other diseases. These shifts in prevalence could be explained by the release of genetic susceptibility for disease in the form of gene-by-environment (GxE) interactions. Yet, the detection of interaction effects requires large sample sizes, little replication has been reported, and a few studies have demonstrated environmental effects only after summing the risk of GWAS alleles into genetic risk scores (GRSxE). We performed extensive simulations of a quantitative trait controlled by 2500 causal variants to inspect the feasibility to detect gene-by-environment interactions in the context of GWAS. The simulated individuals were assigned either to an ancestral or a modern setting that alters the phenotype by increasing the effect size by 1.05–2-fold at a varying fraction of perturbed SNPs (from 1 to 20%). We report two main results. First, for a wide range of realistic scenarios, highly significant GRSxE is detected despite the absence of individual genotype GxE evidence at the contributing loci. Second, an increase in phenotypic variance after environmental perturbation reduces the power to discover susceptibility variants by GWAS in mixed cohorts with individuals from both ancestral and modern environments. We conclude that a pervasive presence of gene-by-environment effects can remain hidden even though it contributes to the genetic architecture of complex traits.

## Introduction

Diseases such as diabetes, cardiovascular disease, and obesity have become highly prevalent in the developed world in a period of just a few generations. For example, more than one third of U.S Citizens are obese (Ogden et al., 2007). The incidence of these “modern” diseases is now also rising in developing countries (Abegunde et al., 2007). Recent changes in lifestyle are thought to be the main drivers of the emergence of these diseases, because genetic changes at the population level only occur after many generations.

Paradoxically, the rapid increase in prevalence of these diseases coincides with large heritability values. There is increasing evidence that the heritability of several traits has increased in the last 50 years. Obesity serves to illustrate this point. An analysis of Swedish military conscripts born from 1951 to 1983 showed an increase in the heritability along with a marked increase in the genetic variance for obesity (Rokholm et al., 2011b). A further study of Danish twins showed that one percentage point increase in the prevalence of obesity accompanies a ~3.3% increase in the genetic variance for the trait (Rokholm et al., 2011a). Thus, the increased influence of the current “obesogenic” environment exerts its effects through a large alteration in the overall contribution of genetic factors to the susceptibility for obesity. The two most likely explanations for this phenomenon consist of (i) uncovering of new cryptic susceptibility variants that did not previously participate in the genetic architecture of the trait (Gibson and Dworkin, 2004), or (ii) an increase in the effect size of variants already associated with obesity before the emergence of the current “obesogenic” environment (Hermisson and Wagner, 2004).

In the last 5 years, thanks to the detection of genetic variants robustly associated by GWAS, the presence of gene-by-environment interactions (GxE) has been confirmed for several traits. However, the discovered GxE effects explain just a minor fraction of variance, suggesting that most interaction effects remain hidden. The poor availability of reliable environmental data constitutes one the major hurdles to detect GxE interactions. Genetic variation of common nature can be interrogated systematically with commercial genotyping arrays, but the availability of counterpart environmental information is often patchy and inconsistent, impeding a systematic interrogation of GxE effects (Patel et al., 2010, 2013). Moreover, the lack of high-throughput environmental data makes it difficult to replicate consistently GxE findings across datasets (Patel and Ioannidis, 2014). A second obstacle lies in the large sample size that is required to discover interaction effects univocally. For example, an early report observed that physical activity and diet modulate the effects of FTO variants on obesity (Demerath et al., 2011), but the evidence remained unclear in subsequent studies (Hubacek et al., 2011; Van Vliet-Ostaptchouk et al., 2012) until a large meta-analysis of 45 studies of ~240,000 samples confirmed this interaction. Specifically, this meta-analysis established that the risk effect of FTO alleles was ~100 and 40% larger in physically inactive relative to active individuals from North America and Europe, respectively [Odds Ratio: 1.43 vs. 1.22 and 1.27 vs. 1.21, respectively (Kilpelainen et al., 2011)].

Additionally, synergistic interactions between causal alleles and environmental factors are being detected through genetic risk scores (Franks et al., 2013). The calculation of GRS involves generation of a weighted sum of the risk due to several variants into a single figure, thus overcoming the limitation of statistical power for individual SNPs. For example, the interaction between risk alleles and sugar-sweetened beverage intake has been confirmed by means of a predisposition score for obesity based on 32 GWAS-discovered obesity variants. Specifically, the risk in BMI per 10 risk alleles increased by 77% in individuals with ≥1 serving per day compared to sugary beverage intake <1 serving per month (Qi et al., 2012). Similar examples of GRSxE detection have been described for fried food consumption and adiposity (Qi et al., 2014), cigarette use polygenic risk and neighborhood social cohesion (Meyers et al., 2013) and Western dietary patterns and type 2 diabetes (Qi et al., 2009; Nettleton et al., 2013).

In order to quantify how prevalent this GRS-by-environment (GRSxE) contribution may be, we have performed a simulation study of a quantitative trait under “ancestral” and “modern” environments. Our main aim was to define the range of realistic conditions in which GRSxE interaction effects can be detected in the absence of evidence for individual GxE for the contributing alleles. The environmental perturbation and genetic architecture of the trait are based on recent inferences from human GWAS data. We demonstrate that a wide range of perturbation effects is consistent with current observations from GxE studies, although our investigations also show that these effects may heavily reduce the power to detect causal alleles by GWAS.

## Materials and methods

### Genetic architecture of the simulated trait

We performed simulations of a polygenic quantitative trait to study the feasibility to detect gene-by-environment effects in the context of GWAS studies. We considered a trait partially controlled by genetic variants in the context of a total phenotypic variance of 1 (*V_P_* = 1). In all simulations, we approximate the genetic architecture based on two recent inferences regarding the genetic basis of complex traits in humans. First, the trait is controlled by 2500 causal SNPs of common nature (minor allele frequency >5%). This number of genes resembles the number of susceptibility variants inferred for several complex traits [e.g., from ~1700 to 2900 for myocardial infarction and type 2 diabetes, respectively (Stahl et al., 2012)]. Second, we assign the percentage of variance explained by each causal SNP (genetic variance of the trait, *gv*) based on the inferences from a large meta-analysis on normal height variation (Lango Allen et al., 2010). This study discovered 180 loci associated with height, each explaining from 0.012 to 0.28% of the variance in the trait. The contribution of 701 variants of similar effect size (accounting for 16% of the V_P_) was inferred. We thus assigned the inferred distribution to 701 randomly selected variants from the 2500 simulated SNPs (gathered from Supplementary Table 4 in Lango Allen et al., 2010). Each of the remaining 1799 alleles was assigned to explain 0.012% of the variance. Hence, the 2500 simulated common SNPs individually explain from 0.012 to 0.28% of the variance, and the total genetic component of the trait accounts for 36% of the V_P_ (heritability = 36%). Importantly, note that we assign the allelic effects as a percentage of variance that each SNP explains, with the corollary that the actual effect size per allele will depend on the frequency of the causal allele (see next paragraph).

The number of SNPs and *g* of the trait are fixed. Then, for each simulation we re-assign the effect allele frequencies (EAF) and effect sizes (β) at each of the 2500 causal SNPs. To mimic the ascertainment bias of GWAS arrays, EAF values were drawn from a uniform distribution with boundaries 0.05 and 0.95 [*U*_(0.05,0.95)_]. Genotypes were simulated assuming Hardy-Weinberg equilibrium. For example, for a SNP with EAF = 0.4 in a simulation of 10,000 samples, we would assign a value of 0, 1, and 2 phenotype-increasing alleles to ~1600, 4800, and 3600 individuals, respectively. At this point of each simulation, we know the number of alleles that every individual carries at each site, as well as the total genetic variance each SNP explains. We can then easily calculate the effect size (β) of each SNP. The effect of the *i*^th^ SNP on the trait is given by its contribution to the genetic variance of the trait (Park et al., 2010):

$${\text{gv}}_{i}=2*\beta^{2}*{\text{EAF}}_{i}*(1-{\text{EAF}}_{i})$$

For example, a variant that explains 0.28% of the V_P_ with an effect allele frequency of 0.4 would increase the simulated phenotype by 0, 0.076, and 0.153 in individuals with 0, 1, and 2 causal alleles at that position, respectively. We consider an additive polygenic architecture. Thus, for each simulated individual the effects are added additively per allele copy, and summed independently across all 2500 causal loci. After assigning the effects to all SNPs, the additive genetic variance component (V_A_) equals ~0.36. To achieve the desired phenotypic variance (*V_P_* = 1), we assigned a random environmental component (V_E_) to every individual, drawn from a normal distribution with mean 0 and variance 0.64 (*V_E_* = 1 − V_G_). In summary, we simulated a quantitative trait with heritability 36% that results from the additive gene action over 2500 independent causal SNPs of common frequency.

### Modeling a shift in environment that perturbs the genetic effect sizes

The genetic architecture explained above assumes that all individuals experience the same environment. This study investigates the consequences of a change in the environment that also modifies genetic contributions to disease or traits. Consequently, for convenience we call the baseline situation the “ancestral” environment, and postulate a new “modern” environment in which genetic effects are perturbed at some fraction of the 2500 causal SNPs. We also suppose that in contemporary society, some individuals have a lifestyle more close to the “ancestral” one (simplistically, low caloric intake, high activity) while others have a more “modern” lifestyle (they consume sugary beverages and engage in other obesogenic behaviors). In reality there will be a gradation, but the dichotomous model serves for purposes of illustration of the potential consequences for disease for contemporary societies of the transition to a western lifestyle, that may have induced GxE effects (Gibson, 2009). Specifically, we considered the situation in which some or all individuals in the population live in a new environment that provokes a scaling effect (perturbation) in the genetic effect size at a fraction of the 2500 causal SNPs. Thus, simulated individuals can be classified into two binary “unperturbed” and “perturbed” categories, according to the environment they live in. The ancestral and modern environments aim to model a situation in which the genetic susceptibility to disease may have been altered in modern societies as a consequence of the transition to a western lifestyle (Gibson, 2009), that may have induced GxE through scaling effects. Specifically, the “modern” environment alters the genetic architecture of the trait by causing a multiplication of the effect size (β) by a constant factor (e.g., with a 1.5-fold change, a SNP with β_ANCESTRAL_ = 0.06 transforms to β_MODERN_ = 0.09). The strength of the GxE interaction is proportional to, first, the factor of perturbation and, second, the proportion of SNPs that become perturbed in the “modern” environment. For example, physical activity was shown to attenuate the association between rs9939609 in *FTO* and body mass index (BMI) by ~30 to 95% (Andreasen et al., 2008; Kilpelainen et al., 2011). Another recent study on the interaction of sugar-sweetened beverages and BMI described an increase of 77% in the genetic risk per 10 causal alleles for individuals who drink >1 beverage serving per day, which would translate into an ~8% increment in the effect size per variant under the “modern” environment (Qi et al., 2012). In our simulations, we explored the parameter space that ranges from 5 to 100% increase in the genetic effect size (1.05–2-fold change, respectively). Regarding the proportion of SNPs perturbed, we explored the outcomes after perturbing from a minimum of 1% to a maximum of 20% of the causal variants (25 and 500 of the 2500 simulated SNPs, respectively).

### Selection of SNPs that become perturbed in the “modern” environment

All causal SNPs do not account for the same proportion of genetic variance in the simulated trait. Therefore, the degree of GxE we induce also depends on the actual effect size of the perturbed SNPs. We explored two different models of SNPs that become perturbed. In model 1, the SNPs were chosen at random, whereas in model 2 they were chosen from those explaining most of the variance (e.g., the 250 SNPs with highest explained variance in simulations if 10% of the variants were perturbed). Importantly, the random environmental component (V_E_) was drawn equally in both “ancestral” and “modern” environments. In other words, the “modern” environment induces an increase in the V_P_ after perturbation that is entirely dependent on the genetic component of the trait, thus increasing the V_G_ and heritability. Models entailing an increase in V_E_ could be similarly explored, but we do not do so here. Moreover, we note that although we only simulate scaling effects (at the SNP level), since only a small portion of variant effects is perturbed, there are also rank effects at the phenotype level.

### Three scenarios of SNP discovery in a GWAS setting

**Figure 1:**
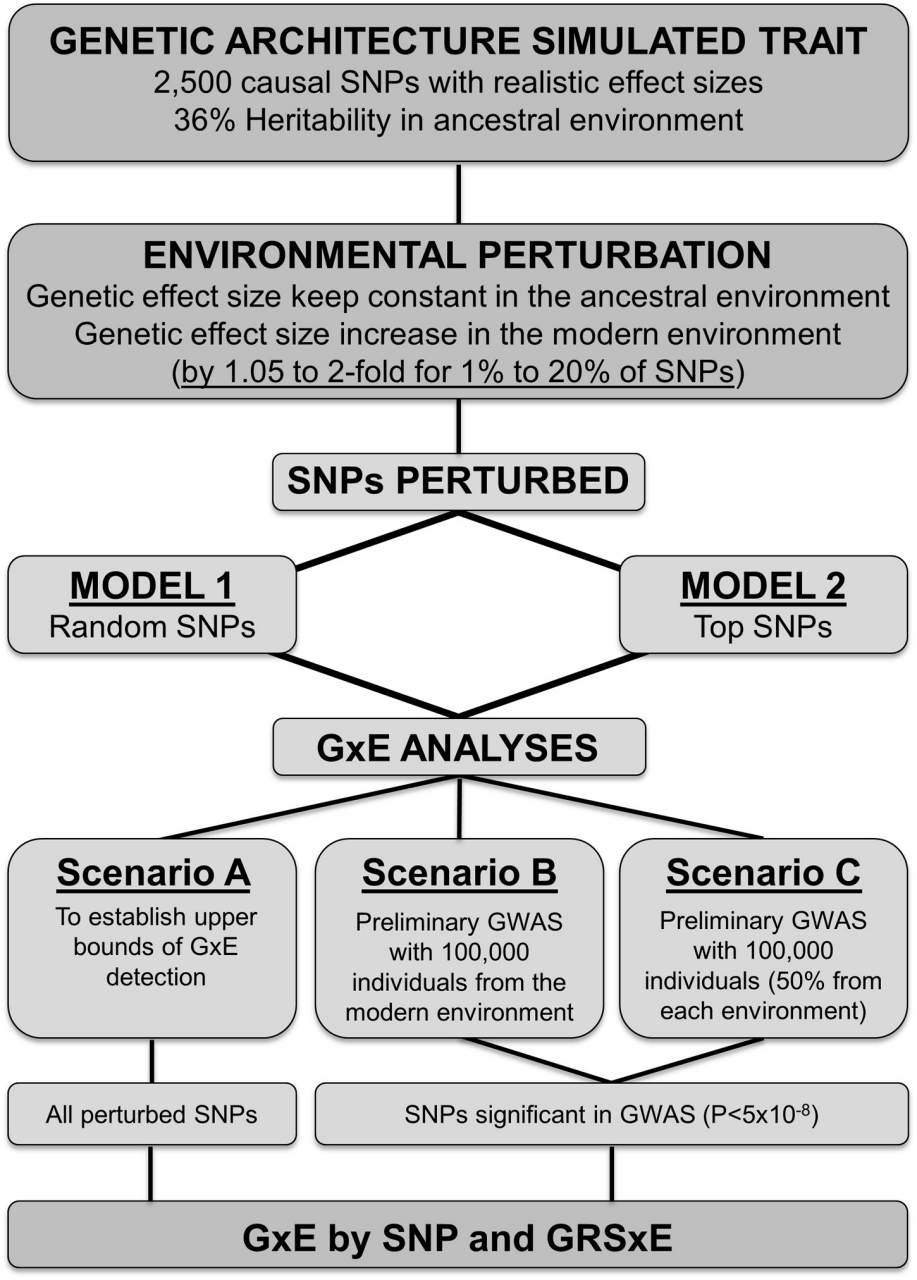
**Summary of the steps followed in the study**.

For both perturbation models 1 and 2 explained above, we set up three different scenarios to perform a “SNP discovery” process to ascertain the variants that were subsequently tested for the presence of GxE effects (see a workflow summary in Figure 1). In the first scenario, “scenario A,” we act as if all perturbed SNPs were known, and forward them directly to GxE analysis (see next section). “Scenario A” avoids the GWAS discovery step and thus constitutes an ideal situation to establish an upper bound for the range of perturbation effects that can be detected under models 1 and 2.

However, in reality we do not know in advance which SNPs may have undergone environmental perturbation in effect size. Usual practice consists on testing GxE effects for variants that have been previously associated by GWAS. To mimic the situation, we developed two further scenarios in which we added a preliminary GWAS step to discover SNPs. In “scenario B,” we performed a GWAS in which 100% of the samples were selected from the “modern” perturbed environment. In “scenario C” we performed GWAS upon a sample that is drawn equally from each of the two environments (50% of the individuals come from the “ancestral” and “modern” settings, respectively). In other words, “scenario C” corresponds to a situation in which half of the society lives in an “ancestral” environment (e.g., extensive physical activity in daily life and low fat diet), whilst the other half follows a “modern” lifestyle that increases the effect size of perturbed alleles. Importantly, we do not “know” which environment each individual lives in, in the sense that this information is not included in the discovery GWAS. For both scenarios, we performed a two-stage genome-wide screen in which the quantitative phenotype is regressed against the allele dosage at each SNP. In the discovery screen, we assay the 2500 simulated SNPs in a sample of 50,000 individuals. SNPs that achieve *P* < 10^−5^ in the discovery GWAS are then assayed in a meta-analysis with 100,000 individuals after joining the 50,000 samples from the discovery GWAS with a new simulated replication sample of 50,000 individuals. Finally, SNPs associated with the quantitative trait at *P* < 5 × 10^−8^ in the meta-analysis are then forwarded to a novel sample of 40,000 individuals for the GxE analysis described in the next section.

### Testing for gene-by-environment effects after perturbation

A central focus of our study lies in the evaluation of the power to detect the GxE effects in our simulated trait. We aimed to evaluate the performance of two different approaches, namely (i) power of detection through the examination of individual SNPs and (ii) by means of unweighted genetic risk scores (GRS) that sum up the number of causal alleles for each individual (without weighting each allele by its effect size). To do so, for each scenario we simulated two cohorts of 20,000 individuals each that are sampled from the “ancestral” and “modern” environments, respectively. For each simulated individual, we know its phenotype, the number of causal alleles at each SNP (coded as “0,” “1,” and “2”), the total number of causal alleles over all selected loci (GRS) and the environment it belongs to (coded as “0” and “1” for “ancestral” and “modern” environments, respectively). In each simulation of 40,000 individuals, we tested the interaction between genetic component and environment by means of a multiple linear regression: Y_j_ = β_0_ + β_G_ * χ_(G)_ + β_E_ * χ_(E)_ + β_(G * E)_ * χ_(GE)_ to estimate the regression coefficient β_(G * E)_, with Y_j_, χ_(G)i_, and χ_(E)i_ recording the phenotype, allele dosage (or GRS) and environment of the individual j, for individuals 1,…, 40,000.

**Figure 2:**
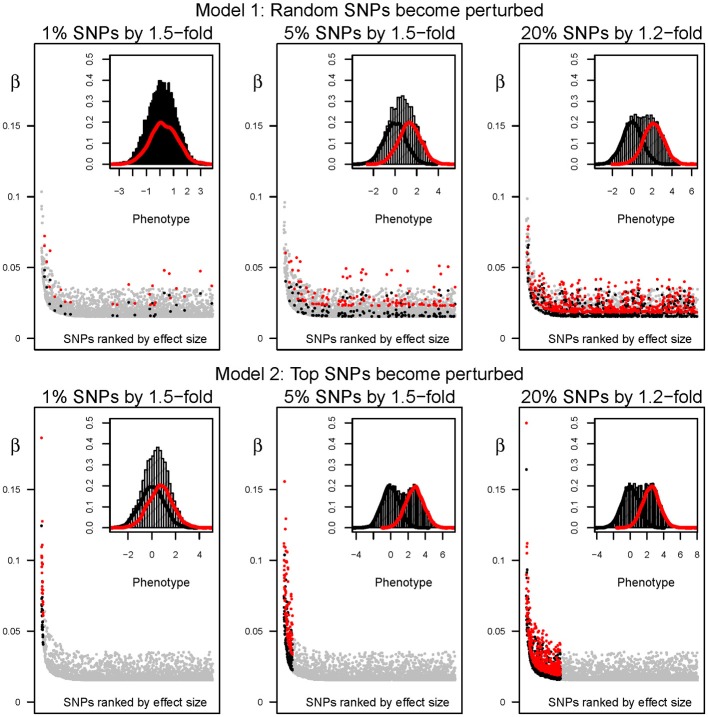
**Distribution of effect sizes and phenotypes before and after perturbation**. From left to right, outcomes after perturbing the effect size of 1, 5, and 20% of the SNPs by 1.5, 1.5, and 1.2-fold, respectively. Top and bottom panels have been simulated under models 1 and 2, respectively. Major plot: the effect size (β) of the 2500 causal SNPs is shown in y-axis. SNPs are ranked in x-axis according to the percentage of variance explained by each variant in the “ancestral” environment (from left to right). Gray dots correspond to the effect size in the “ancestral” environment. The scaling effect among environments is shown in black and red. Specifically, the black and red dots in main panel indicate, respectively, the effect size before and after (in “ancestral” and “modern” environments). Minor plot: distribution of phenotypes in a cohort of 10,000 simulated individuals. Black and red curves show the histogram of phenotypes for the same individuals, before and after perturbation. The curve in the background represents the histogram of phenotypes if the two simulated samples are joined into a cohort of 20,000 individuals.

In summary, we explored two different ways to select SNPs that undergo perturbation and three different procedures to choose the actual variants upon which we test for gene-by-environment interactions. For each of the six resulting combinations (models 1 or 2, and scenarios A, B, or C), we explored 400 combinations of parameters. Specifically, the percentage of SNPs that experienced perturbation ranged from 1 to 20% (20 steps of 1%), and the factor of perturbation ranged from a 1.05–2-fold change in effect size (20 steps of 0.05-fold increments). We performed five different replications for each of the 400 combinations, and thus 2000 simulations for each of the six combinations. Results are summarized as heat maps that interpolate relevant parameters across a continuous range of values (Figures 2, **4**–**7**, and Supplementary Table 1).

### Statistical analysis

All the analyses were performed using the R software v.3.0 (R Core Team, 2013). Associations between the simulated phenotype and allele dosage, as well as the GxE interactions, were tested with the *lm* function. Heatmap plots were generated using the *fields* and *akima* R packages.

## Results

We simulated an environmental perturbation in genetic effect sizes to explore the feasibility of detecting gene-by-environment interactions. In the “ancestral” environment, the 2500 causal variants explained from 0.012 to 0.28% of the phenotypic variance. In the “modern” setting a percentage of variants ranging from 1 to 20% underwent perturbation, and their effect sizes increased by a constant factor that ranged from 1.05 to 2-fold. We applied two different models to select the causal SNPs that become perturbed in the second “modern” environment, and built three scenarios to select the SNPs upon which we investigated the feasibility of detecting gene-by-environment interactions following the workflow in Figure 1. A detailed summary of the results for each simulation is available in Supplementary Table 1.

### Effects of the “modern” environment in the distribution of effect size and phenotypes

The actual effect size of each causal allele depends on the frequency and variance explained by the causal variant. For example, we set the strongest contribution in the “ancestral” environment at ~0.3% of the variance explained. If that allele has a frequency of 0.5, it would present an effect size of 0.075 (β_ANC_), increasing the phenotype by 0, ~0.075 and 0.15 in individuals with zero, one and two causal alleles, respectively. If it becomes perturbed in the “modern” environment by the strongest perturbation possible (2-fold change; β_MOD_ = 2 * β_ANC_), the effect size would increase from ~0.075 to 0.15. Thus, the variant would increase by 4-fold the percentage of phenotypic variance it accounts for, hiking from ~0.3 to 1.2% (see Materials and Methods).

**Figure 3:**
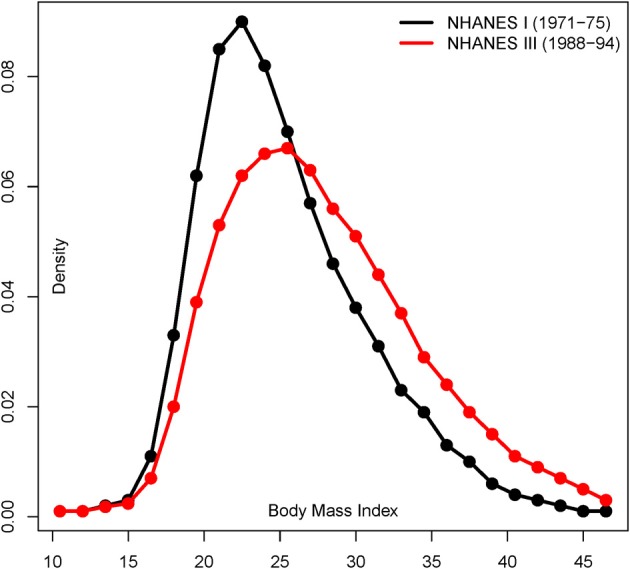
**Shift in BMI in U.S males from 1971–1975 to 1988–1994**. Distribution of BMI in North American males (20–55 age) studied in the NHANES I and III health and nutritional surveys (adapted from Figure 1 in Cutler et al., 2003).

The differences in the distribution of phenotypes under each environment not only depend on the strength but on the proportion of variants that become perturbed in the “modern” setting. The same perturbation inducing a 2-fold increment in the effect size, but acting upon 20% of the SNPs, would result in a distribution of phenotypes that do not overlap extensively. We illustrate the resulting distribution of phenotypes under the “ancestral” and “modern” environments for a range of perturbation effects in Figure 2 (black and red lines, respectively). For instance, the average phenotype under “modern” conditions after perturbing 20% of the causal SNPs by 1.2-fold is two standard deviations above the average phenotype under the “ancestral” environment. Overall, perturbation leads to a flattened distribution of phenotypes when individuals from both environments are combined, and the increase of phenotypic variance is proportional to the percentage of people that live in the “modern” environment. The differences are strengthened under model 2, because the SNPs that already present the largest effect sizes in the “ancestral” environment are chosen for perturbation in the “modern” setting. Indeed, the most extreme simulated perturbation, such as multiplying the effect of 20% of the variants by two, results in bimodal distributions that can be easily distinguished and are probably biologically unrealistic. However, the differences are much subtler for most of the parameter space, and in next sections we refer to the parameter space that results in a change in the distribution of phenotypes that resembles that of typical traits such as contemporary BMI (see Figure 3 for a real example based on the change in BMI shown by North American males).

**Figure 4:**
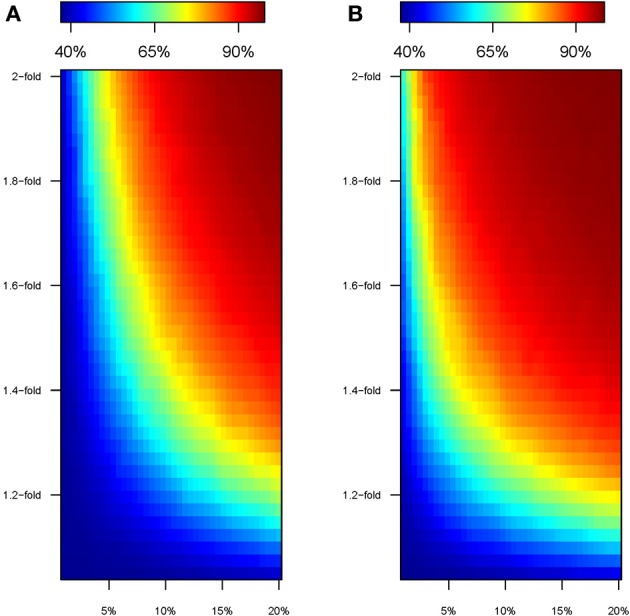
**Heritability of the simulated trait in the “modern” environment**. Color map showing the heritability in cohorts perturbed under model 1 **(A)** and model 2 **(B)**, according to the percentage of SNPs perturbed (x-axis) and the factor of perturbation in effect size (y-axis).

The perturbation in genetic effect sizes prompted by the “modern” environment leads to an increase in the heritability of the quantitative trait (Figure 4). The phenotype presents a basal heritability of 36% in the “ancestral” environment, but it easily boosts in the “modern” setting. For instance, a 1.2-fold increase in the effect size of 20% of the causal SNPs leads to a heritability of ~80%, and a similar effect is achieved with a 1.3 and 1.6-fold change acting upon ~10 and 5% of the causal variants, respectively. This happens because the “modern” environment induces a hike in V_P_ that is entirely due to a higher V_G_ (we keep V_E_ constant, see Material and Methods). Again, the effect is more pronounced under Model 2 (Figure 4B). For instance, 2-fold increments in the effect size inevitably lead to unrealistic heritability values above 90% in the “modern” environment.

We illustrate the effects of the “modern” environment on (i) the genetic effect sizes of perturbed SNPs (major graphs in Figure 2), (ii) the differences in the distribution of phenotypes between the “ancestral” and “modern” lifestyles (small graphs in Figure 2), and (iii) the heritability of the simulated trait (Figure 4). We next describe the ability to detect gene-by-environment interaction effects induced by the “modern” setting. We compare the ability to detect GxE interactions at the SNP level with that of GRSxE analyses. Overall, we consider three different scenarios to ascertain candidate SNPs, and examine for GxE effects in cohorts of 40,000 individuals in which half of the samples come from the “ancestral” and “modern” environments, respectively.

### Detection of GxE effects when all perturbed variants are known (scenario a)

**Figure 5:**
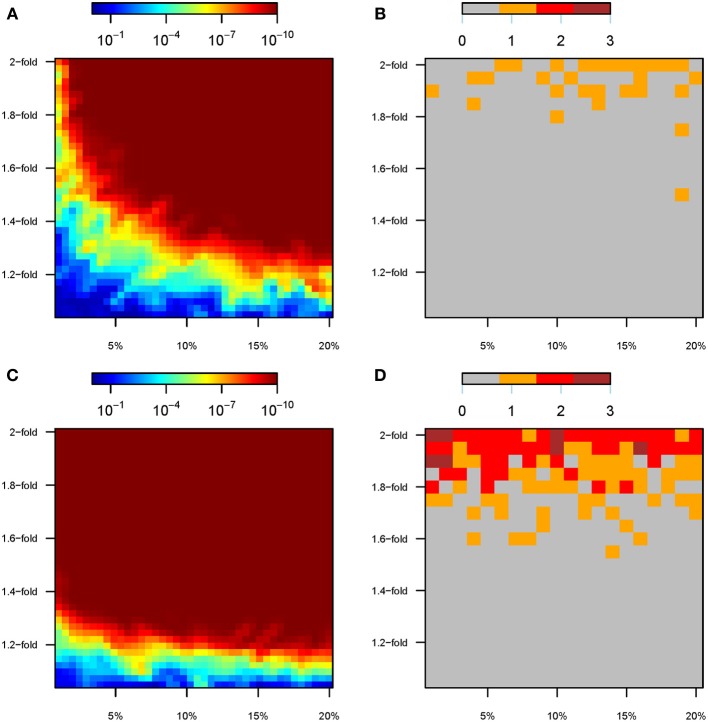
**GxE analyses under scenario A**. For scenario A, color maps showing the results of the gene-by-environment interaction analyses according to the percentage of SNPs perturbed (x-axis) and the factor of perturbation in effect size (y-axis). **(A)** *P*-value of the GRSxE interaction under model 1. **(B)** Number of SNPs at genome-wide significance levels (*P* < 5 × 10^−8^) for GxE under model 1. **(C)** *P*-value of the GRSxE interaction under model 2. **(D)** Number of SNPs at genome-wide significance levels (*P* <5 × 10^−8^) for GxE under model 2. Panels **(B,D)** record the largest number observed out of five permutations.

Even if the analyses include all variants that are perturbed (that is, known from the model, without a GWAS discovery step), GxE effects tend to remain undetected at the SNP level (see Figure 5). Specifically, under Model 1 only 32 out of 2000 simulations (1.6%) achieved genome-wide significance (*P* < 5 × 10^−8^) for any SNP in the GxE analyses, and all of these required a >1.5-fold change in the effect size (Figure 5B). Indeed, at most a single variant was detected in each simulation, even if we tested for GxE individually for all perturbed SNPs (e.g., 500 tests for GxE when 20% of the variants were perturbed). Furthermore, only 14% of the 100 simulations with a 2-fold change in the effect size harbored a variant that passed the threshold for genome-wide significance (Figure 5B). Conversely, there was a wide range of perturbation parameters for which genetic risk scores, the sum of the total number of causal alleles each individual carries, constituted a powerful tool to detect interaction effects induced by the “modern” environment (Figure 5A). For instance, GRSxE interaction terms using GRS calculated over 250 perturbed SNPs (10% of causal variants) showed extremely low *p*-values (*P* < 10^−10^) for all the ranges from 1.3 to 2-fold change in the genetic effect size. Indeed, tiny increments in the effect size, such as a 1.2-fold change, resulted in ~100% of the simulations detecting GRSxE effects at the *P* < 0.05 significance level (notice that we performed a single GRSxE test per simulation, because the allelic count of all tested variants were collapsed into a single number). Only the parameter space correspondent to <1.1-fold changes for <5% of the causal variants consistently resulted in non-significant GRSxE interaction terms (Figure 5A).

The same patterns were observed under the environmental perturbations of Model 2, although an overall increased ability to detect interaction effects was noticed (Figures 5C,D). Specifically, 12.8% of the simulations (255 out of 2000) led to significant GxE effect at the SNP level, although 74.1% of those showed a single variant being genome-wide significant (189 out of 255). It was necessary to perturb genetic effects by 1.8–2-fold to achieve several variants being significant at the SNP level (Figure 5D). The interaction effects induced by the “modern” environment are almost universally detected through GRSxE analyses (Figure 5C).

### Detection of causal alleles by GWAS after modern environmental perturbation

In “scenario A,” the environmental perturbation in effect sizes can be easily detected with GRSxE analyses. These results establish an upper bound for the ability to detect gene-by-environment effects induced by the “modern” lifestyle, because the analyses are restricted to the truly perturbed variants. Yet, for real traits it is uncertain which SNPs may present GxE effects. Usual practice consists of prioritizing variants unequivocally associated to the trait of interest, such as the alleles discovered by GWAS. To mimic this procedure, we perform a preliminary GWAS study to ascertain variants for GxE analyses.

**Figure 6:**
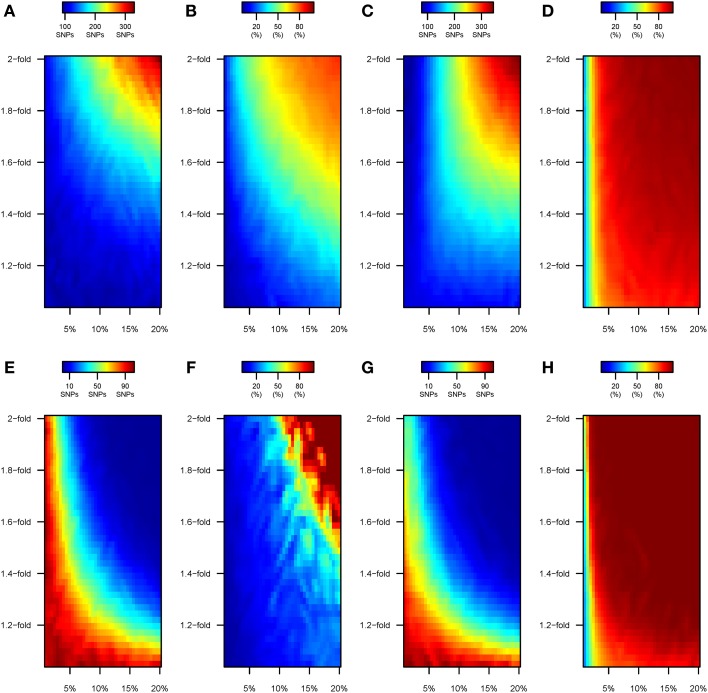
**Number of SNPs discovered by GWAS under scenarios B and C**. Color maps showing the results of the GWAS upon cohorts of 100,000 individuals with (i) 100% of the samples drawn from the “modern” environment (scenario B; top panels, **A–D**) and (ii) 50% of the samples drawn from each “ancestral” and “modern” environments (scenario C; bottom panels, **E–H**). Specifically: **(A,E)** Under model 1, number of variants discovered by GWAS at genome-wide significance levels (*P* < 5 × 10^−8^). **(B,F)** Under model 1, percentage of the genome-wide significant variants that have undergone perturbation. **(C,G)** Under model 2, number of variants discovered by GWAS at genome-wide significance levels (*P* <5 × 10^−8^). **(D,H)** Under model 2, percentage of the genome-wide significant variants that have undergone perturbation.

GWAS meta-analyses of 100,000 individuals entirely drawn from the “ancestral” environment detected ~90 genome-wide significant variants, accounting for ~15% of the heritability (data not shown). GWAS on cohorts with 100% of the individuals being “perturbed” under model 1 led to an increased ability to detect variants associated to the trait (Figure 6A). The number of detected variants oscillated from 100 to 150 for the most realistic range of perturbation parameter space, and hiked to ~300 when GWAS was performed upon 100,000 very heavily “disturbed” individuals (e.g., 2-fold change in the effect size for ~20% of the causal variants). A progressively larger number of the associated variants that are detected correspond to perturbed variants (Figure 6B). The tendency to detect increasing proportions of perturbed variants becomes exacerbated under model 2. Specifically, and even if similar numbers of significant variants are detected by GWAS (Figure 6C), the increment in SNP detection corresponds to perturbed variants (Figure 6D).

Highly divergent patterns were observed when we perform a preliminary GWAS upon a mixed sample of individuals drawn equally from the “ancestral” and “modern” environment (“scenario C”). Under Model 1, the number of variants detected by GWAS still remained close to ~90 only if the 50% of GWAS individuals coming from the “modern” environment had only been perturbed slightly (e.g., <1.2-fold for <5% of the causal SNPs, bottom-left corner in Figure 6E). The ability to detect causal alleles dropped when more extensive perturbations were simulated. For instance, ~60 variants were detected at genome-wide significance levels when 7% of the variants had their effect size multiplied by 1.3-fold, and almost no variants are discovered if the same percentage of SNPs underwent a 1.8-fold change in the effect size, or with a 1.3-fold increase for 20% the causal SNPs. Interestingly, the increasingly reduced number of variants discovered by GWAS under “scenario C” corresponded to perturbed SNPs (top-right corner in Figure 6F). Similar patterns were observed for “scenario C” under model 2 of perturbation (Figures 6G,H). As discussed below, we attribute these effects to the increase in phenotypic variance being greater than the individual genetic effects of each SNP.

### Detection of gene-by-environment interactions with SNPs detected by GWAS

**Figure 7:**
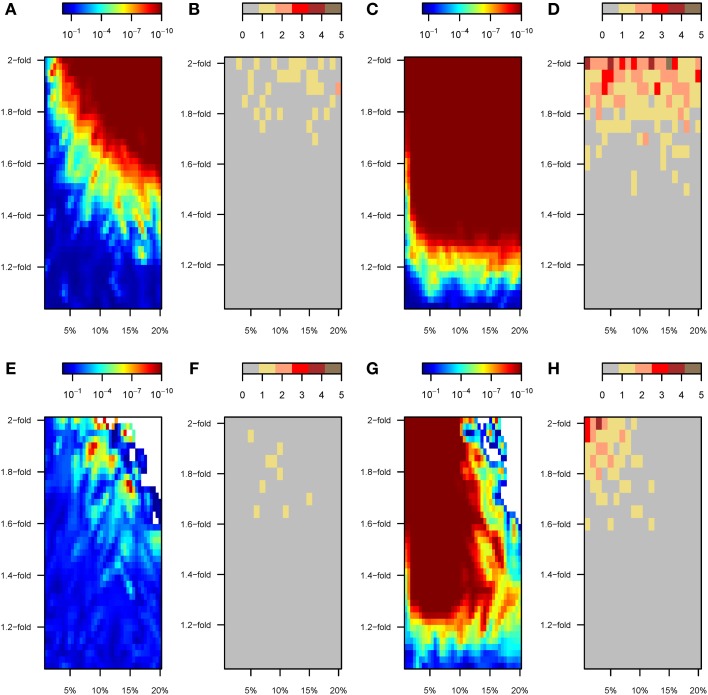
**GxE analyses with SNPs discovered in a preliminary GWAS (scenarios B and C)**. Color maps showing the results of the gene-by-environment interaction analyses according to the percentage of SNPs perturbed (x-axis) and the factor of perturbation in effect size (y-axis). Results for scenario B are shown in top panels **(A–D**). Specifically: **(A)** *P*-value of the GRSxE interaction under model 1. **(B)** Number of SNPs at genome-wide significance levels (*P* < 5 × 10^−8^) for GxE under model 1. **(C)** *P*-value of the GRSxE interaction under model 2. **(D)** Number of SNPs at genome-wide significance levels (*P* < 5 × 10^−8^) for GxE under model 2. The corresponding results for scenario C are shown in bottom panels **(E–H)**. Panels **(B,D,F,H)** record the largest number observed out of five permutations. White areas in top right corners in panels **(E,G)** correspond to parameter space with no SNPs detected by GWAS and thus missing GRSxE analyses.

The enhanced power to discover SNPs under “scenario B” resulted in patterns of GxE interaction detection that are similar to those observed for “scenario A,” in which only perturbed variants were used (Figures 7A–D). SNP-by-SNP tests rarely resulted in significant GxE interaction coefficients (Figure 7B). By contrast, a wide range of the parameter space led to significant GRSxE evaluations, starting from ~1.4-fold change for ~5% of the variants to any stronger perturbation, Figure 7A). Similarly, under model 2 the tendency toward significant GRSxE detection was exacerbated (Figure 7C), and GRSxE interactions were significant for the whole range of simulated parameters. In these analyses, only GWAS performed upon strongly perturbed individuals (1.8–2-fold change in β) permitted detection of perturbed SNPs that were consistently significant at the individual level in the GxE analysis (Figure 7D).

A reversed pattern was observed under “scenario C.” The proportion of perturbed SNPs among the detected variants was higher as perturbation strengthened, but it became negligible in absolute terms because almost no variants were detected by GWAS. Thus, the overall poor performance of mixed GWAS to detect perturbed SNPs rendered almost impossible the task of detecting GxE effects with GWAS SNPs, even at the GRSxE level (Figures 7E,F). The compromised detection power under “scenario C” does not however preclude the detection of gene-by-environment effects through GRSxE analyses under model 2 (Figures 7G,H).

## Discussion

In this study we performed a series of simulations to inquire under what conditions gene-by-environment effects can be detected. We applied an environmental perturbation upon cohorts of individuals that live in either an “ancestral” environment, or a “modern” setting that leads to an increment in the genetic effect sizes of a percentage of the causal alleles. For a wide range of the explored parameter space, gene-by-environment effects mostly remain unnoticed when interaction is examined at the SNP level. Conversely, GxE analyses are well powered to detect significant interactions when the genetic component of each individual is summarized through genetic risk scores (GRS) that sum up the total number of causal alleles in a single figure. Moreover, we find that the ability to detect perturbed SNPs in a GWAS preliminary to the GxE analysis depends on the mixture of samples coming from each environment. Genome-wide screens performed upon homogeneous cohorts of perturbed individuals show increased power to detect significant gene-by-environment interaction effects. In contrast, GWAS upon heterogeneous mixtures of “unperturbed” and “perturbed” individuals present a decreased ability to detect significant SNPs, thus inhibiting the task of detecting GxE effects.

### Feasibility of the environmental perturbation under the “modern” environment

The validity of the insights gained from this study depends on the plausibility of our model of environmental perturbation, and the extent to which we mimic the reality faced by current GWAS studies. Certainly, it is difficult to evaluate the consequences of the “modern” perturbation in the case of actual human phenotypes because the heritability and phenotype distributions correspondent to the “ancestral” lifestyle are unknown. However, there is increasing evidence that the switch to a western lifestyle may have been coupled with a change in the genetic effects of causal alleles (Gibson, 2009). Human complex traits result from the assemblage of multiple physiological dimensions, which may lead to a canalization of phenotypes whereby genetic effects are minimized following long-term stabilizing selection (McGrath et al., 2011). Under such a theoretical model, the “modern” human standard of living may have uncovered the activity of previously silent, or almost silent, cryptic genetic variability (Hermisson and Wagner, 2004). For example, this could have been the case for polymorphisms lying in genes that participate in pathways involved in neural regulation of appetite (Heber and Carpenter, 2011). These variants may have played a small role in the genetic etiology of weight throughout the history of our species, but may explain a larger proportion of the individual susceptibility to obesity in the modern environment of unrestricted access to processed food. A variety of other similar situations could be imagined, such as the interplay between addiction, tobacco use and lung cancer (Amos et al., 2008). In our simulations, we explore a range of parameter space in which the “modern” environment perturbs from 1 to 20% of the causal variants. Such a change can be easily framed in a pathway perspective. Specifically, one or several physiological pathways participating in the genetic architecture of complex traits may respond differently under the “modern” environment. In the context of a common disease, the environmental perturbation we explore would plausibly amount to an increase in the proportion of the population at risk (as in Figure 3 for real BMI).

Our model postulates one of the simplest instances of GxE in which individuals are assigned to a binary environmental state that would roughly correspond to “ancestral” and “modern” lifestyles. A more realistic scenario of environmental perturbation should summarize the varying fraction of “modern lifestyle” followed by each person into an individual-specific measure, or “exposome” (Patel and Ioannidis, 2014). More complex simulations could be tuned to incorporate more realistic settings. For instance, the extent of exposure to modern lifestyle could be more finely determined (e.g., degree of sedentary behavior, diet patterns, stress at work…) to explore threshold-dependent models of GxE. Our simulations are necessarily a simplification of the almost infinite array of GxE interactions that could arise in the presence of multi-layered and continuous environments that can perturb the genetic effects of causal variants (Luan et al., 2001; Wong et al., 2003). However, the qualitative environmental states in our simulations resemble the practice of recent studies that have confirmed GxE effects after categorizing the environment into binary categories, as has been the case for example in studies of sugar-sweetened beverage consumption and overall diet patterns (Do et al., 2011; Qi et al., 2012).

In addition to the mechanism of perturbation and the binary nature of the simulated environment, the realism of our perturbation model also depends on the likelihood that the explored parameter space is realistic. We chose to approximate this by checking whether the range of simulated effects results in phenotypic distributions that approximate real observations. In the context of BMI, for instance, western urban women have been shown to present an average BMI value that is ~4 standard deviations larger than the corresponding figure for Hadza hunter-gatherer women (see Table 1 in Pontzer et al., 2012). These differences are similar to the average horizontal shift between “ancestral” and “modern” environment that we observe in our simulations (e.g., depending on the percentage of perturbed SNPs, changes in effect sizes by <1.4-fold lead to ~1 to 4 standard deviations of difference in the average phenotype). Furthermore, we also examined the shape of the phenotype distributions. Indeed, we observe significant GRSxE analyses for simulations with parameter combinations that result into more flattened but unimodal distributions of phenotypes, such as those observed in U.S men (Figure 3). Nonetheless, the actual phenotypic variance of a combined population depends on the mixture proportions and even extreme situations in which half of the individuals are raised in each environment do not lead to a bimodal phenotypic distribution in a combined simulation population. The heritability of the trait is also kept within a reasonable range. It can severely hike to 90% in the context of the most severe perturbations, but the actual heritability would lie from 36 to 80% according to the exact proportion of “unperturbed” and “perturbed” individuals.

### Detection of gene-by-environment effects with genetic risk scores

We observe a substantial parameter space in which gene-by-environment effects can be easily detected with genetic risk scores while remaining hidden in individual SNP analyses, even after testing exclusively those variants that were detected in populations perturbed by the “modern environment.” SNP-by-SNP analyses provide anecdotal evidence for significant GxE, and only when extreme perturbations are assayed (e.g., >400 SNPs perturbed by 2-fold in the effect size are necessary to detect a single genome-wide significant variant). Conversely, GRSxE analyses are always significant when β-s are multiplied by 1.3-fold or more, or for the whole range of perturbation parameters when the “modern” environment affects the SNPs that explain most of the variance in the trait (i.e., model 2). These results confirm that a widespread presence of GxE effects is not at odds with the lack of evidence when individual variants are assayed, despite of a substantial presence of interaction effects.

An important aspect of our simulations lies in the choice of variants that are perturbed by the “modern” environment. We observe that it is easier to detect GxE effects when the variants that are perturbed coincide with the alleles that explain most of the genetic basis of the trait, as in model 2. This makes sense considering that these perturbed variants not only present the largest effect sizes, but also have multiplied it in the “modern” environment. The same mechanism explains the increment in the number of variants detected by GWAS when the genome-wide screen is performed entirely upon perturbed individuals, as in “scenario B.” For real traits with widespread GxE effects, it may be key to perform GWAS selecting for perturbed individuals. The selection of those individuals following a “modern” lifestyle would unravel specific pathways that respond badly in face of perturbation, thus enabling a more detailed understanding of the etiology of the diseases of affluence. Nonetheless, it may be inherently complex to design “perturbed-only” GWAS, owing to the difficulty in defining what exactly constitutes the perturbed environment. The sampling of individuals could also be confounded by the fraction of cases that are entirely due to purely environmental causes without any major role of gene-by-environment interactions linked to “modern” life.

### Mixture of environments compromises GWAS discovery power

**Figure 8:**
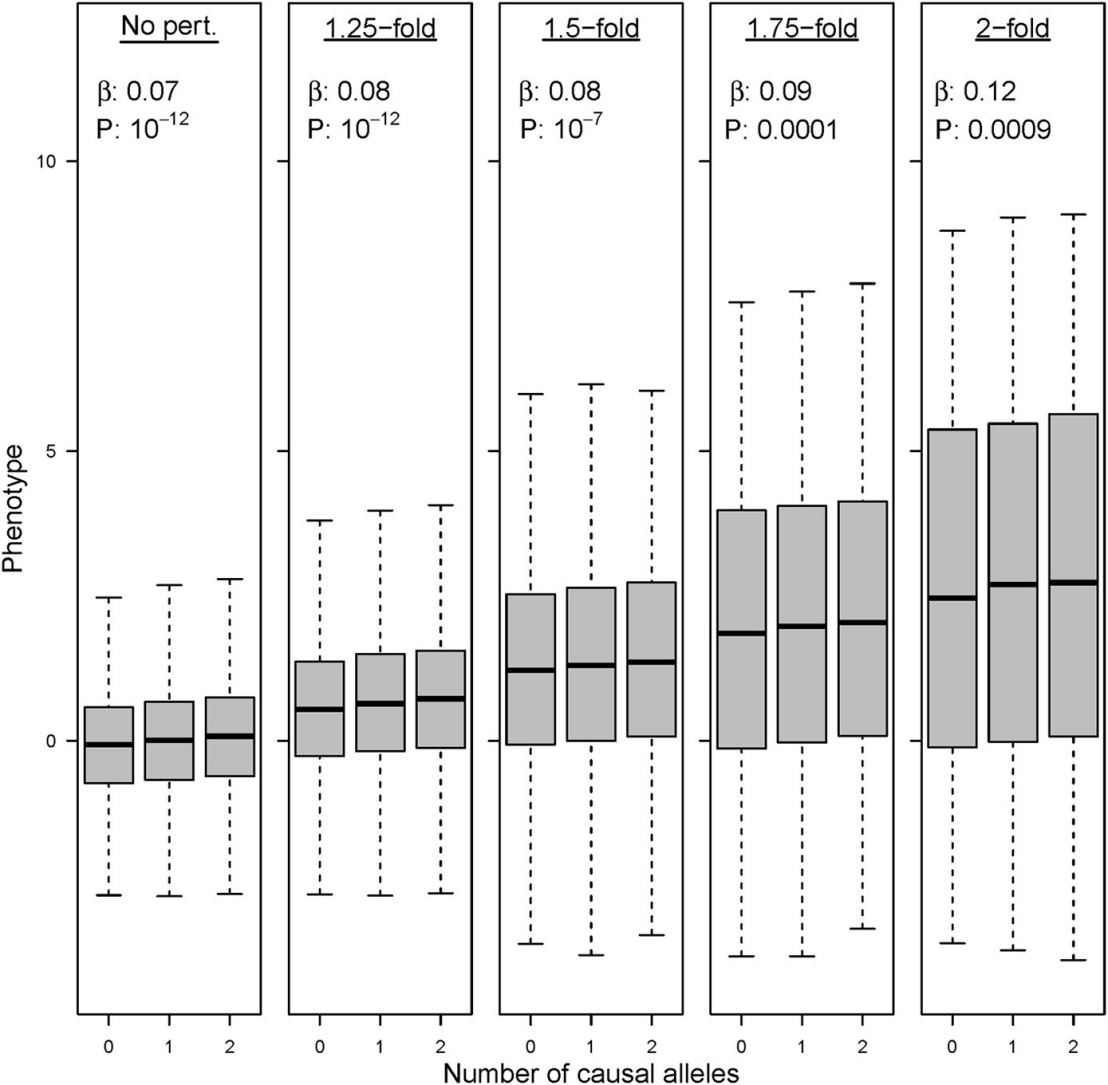
**Environmental perturbation in genetic effect sizes decreases the power of GWAS**. Association results and *P*-value for the same variant under five different GWAS with 20,000 individuals. Left boxplot: a variant explaining 0.3% of the phenotypic variance achieves genome-wide significance in a GWAS with 100% of the samples being drawn from the “ancestral” environment. Successive boxplots: the same variant drops in statistical significance when tested in GWAS in which the allele has undergone a 1.25, 1.5, 1.75, and 2-fold perturbations in 50% of the individuals.

The simulations in which the preliminary GWAS is performed upon cohorts with a mixed environmental exposure (“scenario C”) show a remarkable trend regarding SNP discovery. The combination of “ancestral” and “modern” environments does not compromise the detection of causal variants when perturbation effects are tiny or restricted to a small fraction of the causal SNPs. However, larger perturbations decrease the ability to detect new variants, and statistical power eventually collapses for the strongest range of effects in our simulations. This result makes sense because gene-by-environment interactions add variance and heterogeneity in the estimates of SNP effects. We show the results for a causal variant that explains 0.3% of the variance in an “ancestral” population (Figure 8). This allele achieves *P* < 10^−12^ when assayed in a GWAS with 20,000 individuals that follow the “ancestral” lifestyle. In contrast, the significance worsens (*P* < 10^−7^) when this variant is assayed upon a mixture of 10,000 “ancestral” individuals and 10,000 individuals in which 10% of the SNPs have increased their effect size by 1.5-fold. Eventually, the variant remains completely unnoticed in a mixed GWAS when the effect size increases by 2-fold in the individuals following “modern” lifestyle (P~10^−4^). As a consequence, these variants are not found among the top candidate list in our simulated meta-analysis GWAS.

It is difficult to evaluate the extent to which pervasive gene-by-environment effects have compromised the power to discover associated variants by GWAS. The number of discovered variants correlates with sample size (Visscher et al., 2012), but some other differences among studies can be remarked upon. For instance, a large meta-analysis of ~180,000 individuals reported 180 different loci associated to height, whereas a similarly powered study with >250,000 individuals only described 32 loci for BMI (Lango Allen et al., 2010; Speliotes et al., 2010). This may be explained simply by a difference of narrow sense heritability. On the other hand, the SNP-based heritability in these studies explains a notably greater proportion of the total heritability for height, implying a reduced missing heritability concern. We propose that this difference might be attributed to environmentally-induced heterogeneities in genetic effect size being more prevalent in the case of BMI, in turn explaining the lack of power to detect obesity-related loci. Arguably, this limitation can be avoided in real GWAS through the inclusion of covariates (e.g., variables that capture nutrition and physical activity levels per individual in a GWAS for obesity). However, the potential covariates to be included are often unknown or not available for all the cohorts, as in for example the largest meta-analyses for height and BMI (Lango Allen et al., 2010; Speliotes et al., 2010).

We explore a genetic architecture and a range of perturbation parameters that are based on empirical data, which strengthens the validity of our observations. However, the present study is not devoid of weaknesses. Among others, we have used the same sample size in all the simulated GWAS and GxE studies. This comes at a price, since the range of perturbations that result in significant GRSxE would certainly change if larger studies were performed. Second, we performed simulations of random mating populations with genotypic proportions following strict Hardy-Weinberg equilibrium (HWE). This procedure follows the usual practice consisting of screening polymorphisms for HWE. Nonetheless, confounding of population structure with environmental variability, further complicating the detection of GxE in real studies, remains a possibility. Third, we explored the presence of interactions through unweighted GRS that do not take into account the effect size of each variant. Since only a few variants present notably large effects (Figure 2), in reality weighted and unweighted risk scores are very highly correlated once more than a few dozen loci are incorporated, which minimizes the loss of power to detect GRSxE effects compared to weighted risk scores. Finally, it should be noted that we only simulate causal variants instead of tagging SNPs, which effectively over-estimates effect sizes relative to those discovered in true GRS.

In summary, the present study constitutes a preliminary evaluation of a realistic mechanism by which gene-by-environment interactions may have altered the genetic etiology of human traits. A widespread presence of realistic GxE effects could only be detected by genetic risk scores calculated upon all variants discovered by GWAS. The extent to which these effects have shaped real human traits remains as an open question, and should be studied in future research.

## Author contributions

GG conceived the original idea. UMM and GG designed the study. UMM performed the simulations. UMM and GG interpreted the data and wrote the paper.

## Conflict of interest statement

The authors declare that the research was conducted in the absence of any commercial or financial relationships that could be construed as a potential conflict of interest.
